# Telitacicept as a novel B cell-targeted therapy in autoimmune encephalitis: a case report

**DOI:** 10.3389/fimmu.2025.1640790

**Published:** 2025-09-12

**Authors:** Zhishan Jiang, Huafeng Liang, Sheng Chen, Qinming Zhou

**Affiliations:** ^1^ Department of Neurology, Wuxi branch of Ruijin Hospital Shanghai Jiao Tong University School of Medicine, Wuxi, China; ^2^ Department of Neurology and Institute of Neurology, Ruijin Hospital, Shanghai Jiao Tong University School of Medicine, Shanghai, China; ^3^ Shanghai Jiao Tong University School of Medicine, Shanghai, China; ^4^ Co-innovation center of neurodegeneration, Nantong University, Nantong, China; ^5^ Clinical Center for Rare Diseases, Ruijin Hospital, Shanghai Jiao Tong University School of Medicine, Shanghai, China

**Keywords:** Telitacicept, autoimmune encephalitis, plasma cell, ^18^F-DPA714 PET/MRI, treatment

## Abstract

Autoimmune encephalitis (AE) comprises immune-mediated neuroinflammatory disorders presenting diverse neuropsychiatric symptoms and antibody-specific manifestations. Despite standard immunotherapy, residual disability, treatment intolerance, and relapse risks highlight unmet clinical needs. Telitacicept, a dual target fusion protein that inhibits B-lymphocyte stimulator (BLyS) and a proliferation-inducing ligand (APRIL), suppresses pathogenic B cell activation and autoantibody production, presenting a mechanism-driven therapeutic potential for AE management. Three AE cases with distinct therapeutic complexities are detailed in our study: (1) An anti-N-methyl-D-aspartate receptor (NMDAR) antibody-positive patient experienced recurrent relapses and was a comorbid individual with upper gastrointestinal bleeding. (2) An anti-leucine-rich glioma inactivated 1 (LGI1) antibody patient resisted corticosteroids, intravenous immunoglobulin, and ofatumumab treatment. (3) A newly diagnosed, anti-LGI1 antibody and anti-contactin-associated protein 2 (CASPR2) antibody dual-positive patient required sequential therapy to consolidate the remission and facilitate prednisone tapering. Telitacicept administration achieved symptom remission across all cases, accompanied by reduced antibody titers and stable outcomes over six months. Our case series evaluates the use of telitacicept in AE patients with varied antibody subtypes, particularly for patients with relapsed or refractory disease, intolerance to CD20-targeted agents, or steroid-related complications. Moreover, telitacicept may serve as an effective sequential therapy to sustain remission and reduce long-term steroid dependency.

## 1 Introduction

Autoimmune encephalitis (AE) represents a heterogeneous group of immune-mediated neuroinflammatory conditions, characterized by a multidimensional phenotype, including acute or subacute onset of psychiatric abnormality, cognitive impairment, memory deficit, seizure, speech disturbance, motor dysfunction, consciousness disturbance, autonomic dysregulation, among others (1).

The clinical management of AE involves a stratified immunotherapeutic protocol. First-line treatments include corticosteroids, intravenous immunoglobulin (IVIG), and plasma exchange (PE), while second-line therapies consist of agents such as rituximab (RTX) and cyclophosphamide. Maintenance therapy features prolonged-use agents such as mycophenolate mofetil (MMF), azathioprine (AZA), or repeated RTX.

However, a subset of patients demonstrates suboptimal responses to conventional immunotherapy, manifesting as persistent symptoms or clinical relapses in the follow-up (2). Furthermore, treatment discontinuation is frequently unavoidable, not only due to intolerable adverse reactions but also refusal of long-term steroid use by some patients (1). These persistent therapeutic challenges highlight the critical limitations in current clinical management strategies.

B cells and plasma cells serve as the sources of pathogenic autoantibodies in AE, establishing B-cell-targeted therapies as a promising therapeutic avenue. Anti-CD20 monoclonal antibodies—including RTX and ofatumumab (OFA) —directly deplete B cells, demonstrating inspiring efficacy in patients unresponsive to first-line immunotherapies (2, 3). However, CD20-targeted agents spare plasma cells that actively engaged in antibody secretion and relapse processes. This mechanistic gap accounts for persistent relapse risks and treatment resistance observed in a subset of AE patients undergoing anti-CD20 therapy (4, 5).

Telitacicept, a novel bispecific fusion protein originally developed for systemic lupus erythematosus (SLE), simultaneously inhibits B lymphocyte stimulator (BLyS) and a proliferation-inducing ligand (APRIL), two cytokines regulating B cell and plasma cell maturation. BLyS primarily governs B cell development, while APRIL sustains plasma cell survival and antibody secretion (6). Telitacicept leverages this dual mechanism to suppress pathogenic B cell activation and autoantibody production, demonstrating therapeutic promise for B-cell-mediated autoimmune disorders, including AE (7). Clinical trials in systemic lupus erythematosus (SLE) have demonstrated significant reductions in disease activity (8), and emerging data in myasthenia gravis suggest favorable efficacy (9, 10). Collectively, these data suggest the potential efficacy of telitacicept in AE.

To date, only a single documented case report described the effectiveness of telitacicept as sequential therapy in a case of overlapping syndrome of myelin oligodendrocyte glycoprotein antibody-associated disease (MOGAD) and anti-N-methyl-D-aspartate receptor (NMDAR) encephalitis (11). Here, we present the first cases in three AE patients with varied antibody subtypes and heterogeneous disease profiles, including those refractory to first-line and CD20-targeted therapy and cases requiring steroid-sparing approaches. This extends the application of telitacicept in clinical practice and provides preliminary real-world evidence of its therapeutic efficacy and acceptable safety profile in AE management.

## 2 Case presentation

### 2.1 Case 1

A 56-year-old male visited our hospital for psychiatric symptoms and cognitive decline over two months. Cerebrospinal fluid (CSF) analysis revealed elevated white cell count (6×10^6^/L) and protein (923.98 mg/L) with normal glucose and chloride levels. AE antibodies were tested using the cell-based assay (CBA) method at Simcere Diagnosis (Nanjing, China). Anti-NMDAR antibody was detected in CSF at a 1:10 titer, confirming the diagnosis of anti-NMDAR encephalitis. His modified Rankin Scale (mRS) score was 3 and his Clinical Assessment of Autoimmune Encephalitis (CASE) score was 3. Additionally, his Mini-Mental State Examination (MMSE) score was 13, revealing moderate cognitive impairment. First-line immunotherapy with IVIG (0.4 g/kg/d) for five days and intravenous methylprednisolone pulse (IVMP) was given immediately after diagnosis and achieved marked remission. Still, it was complicated by upper gastrointestinal bleeding, thrombosis in the intermuscular veins of the left calf and severe left lower extremity arterial stenosis. Maintenance therapy with tapering oral prednisone and MMF (0.5 g twice daily) were instituted.

Despite maintenance immunosuppression, his psychiatric symptoms reoccurred 5 months after discharge. His CASE score rose from 1 to 3 and MMSE score dropped from 23 to 18. Anti-NMDAR antibody was positive in CSF with a titer of 1:1. Repeated IVIG and IVMP were given yet the patient showed little improvement. Therefore, off-label use of OFA (20mg per week for 2 weeks) was given, achieving significant symptomatic improvement.

Although additional doses of OFA (20mg per 4 weeks for 8 weeks) were given subsequently, the patient experienced the third disease episode 7 months after the second discharge, evidenced by a worsening mRS score of 2. The patient refused lumbar puncture, so serum anti-NMDAR antibody was detected revealing a titer of 1:100. The patient once again had OFA administration (20mg per week for 2 weeks) which induced profound B cell depletion (CD19^+^ 0.3%, CD20^+^ 0%). The symptoms improved slightly, but the patient still had prolonged mental disorders and sensory abnormalities.

**Figure 1:**
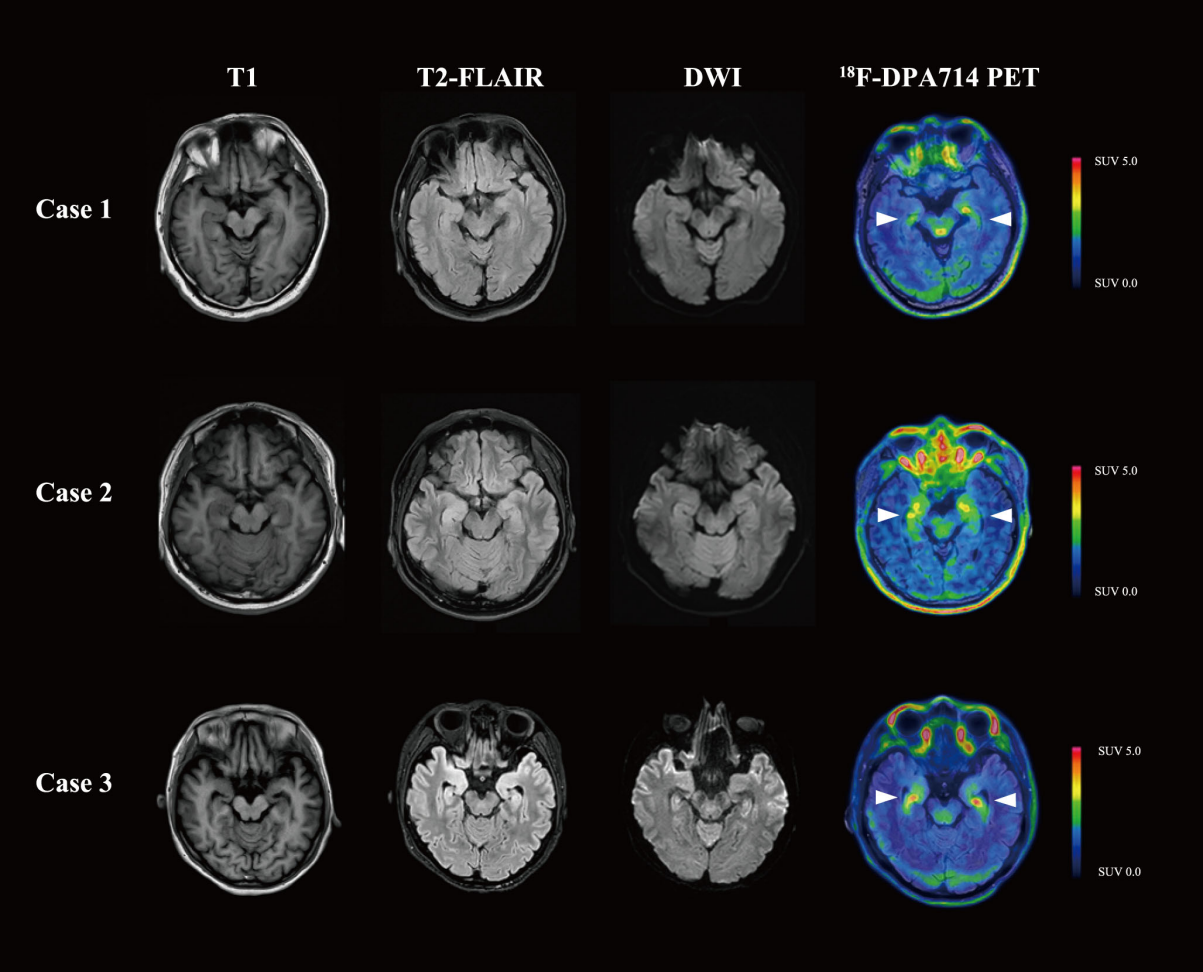
Brain MRI and ^18^F-DPA714 PET/MRI images of the three patients. Cases 1 and 2 presented with normal MRI, while Case 3 was found to have T2-FLAIR and DWI hyperintensity in the bilateral medial temporal lobes. In ^18^F-DPA714 PET/MRI, all three patients had elevated uptake in the bilateral medial temporal lobes. T1, T1-weighted imaging; T2-FLAIR, T2-weighted fluid attenuated inversion recovery; DWI, diffusion weighted imaging.

The fourth disease episode developed 3 months after the previous relapse. Upon readmission for evaluation, neurological evaluation demonstrated aggravated cognitive deficit and memory impairment, with mRS score of 2, CASE score of 4, and MMSE score of 17. Anti-NMDAR antibody was constantly positive in serum (titer 1:100). However, the laboratory tests revealed normal serum IgG (7.89 g/L) and IgM (0.51 g/L), as well as a depleted level of CD20^+^ B cell (0%) and CD19+ B cell (0.1%). While cranial magnetic resonance imaging (MRI) revealed no significant abnormalities, ^18^F-DPA714 PET/MRI imaging demonstrated elevated tracer uptake in the bilateral medial temporal lobes (**Figure 1**) (11). Given the patient’s history of multiple relapses and treatment-refractory disease, along with comorbid conditions, telitacicept (160 mg per two weeks for 12 weeks) was added to the ongoing regimen of oral prednisone (5 mg once daily) and MMF (0.25 g twice daily). Laboratory monitoring conducted 3 months after telitacicept initiation revealed a slight decline in serum immunoglobulins (IgG 6.2 g/L, IgM 0.40 g/L) concomitant with serological anti-NMDAR antibody reduction (titer 1:10). Clinical improvement became perceptible one month after telitacicept initiation and was evidenced by affective stabilization and cognitive enhancement, with an mRS score of 1, a CASE score of 2 and an MMSE score of 24. Longitudinal monitoring over 6 months demonstrated sustained therapeutic efficacy, with neurological function preservation reflected in both stable mRS and CASE assessment at 1. No disease flares or treatment-emergent adverse events were documented during this period.

### 2.2 Case 2

A 42-year-old man presented with progressive memory deficits over one month, accompanied by left upper–limb myoclonic jerks. On admission, neurological examination revealed speech disfluency and short-term memory impairment, with an mRS score of 2, CASE score of 5, and an MMSE score of 20. CSF analysis revealed normal opening pressure, cell count, proteins, glucose, and chloride levels. AE antibodies test (CBA, Simcere Diagnosis, Nanjing, China) indicated anti-leucine-rich glioma inactivated 1 (LGI1) antibody was positive in CSF (titer 1:10) and serum (titer 1:1000). Ambulatory electroencephalogram indicated sharp waves in the temporo-centro-occipital area. While Cranial MRI and whole-body ^18^F-FDG-PET/CT were normal, cranial ^18^F-DPA714-PET/MRI revealed an elevated neuroinflammation in bilateral medial temporal lobes (**Figure 1**). Therefore, the patient was diagnosed with anti-LGI1 antibody encephalitis.

First-line immunotherapy with IVIG (0.4 g/kg/day) and IVMP therapy failed to improve symptoms, with a persistent mRS score of 2 and MMSE score of 20. Subsequent OFA induction (20 mg per week for 3 weeks) achieved complete CD20^+^ B cell depletion from baseline 15.8% to 0%, accompanied by serological anti-LGI1 antibody clearance. Maintenance therapy with tapering oral prednisone and MMF was given for 12 months. During the 6-months follow-up the patient remained clinically stable with an mRS score of 1, a CASE score of 3, and an MMSE score of 23.

Eighteen months after discharge, the patient relapsed with memory impairment, altered episodes of agitation and depression, and sleep disturbance. At readmission, his mRS score was 2, CASE score was 5, and MMSE score was 20. Serum anti‐LGI1 antibody titers had re-emerged at 1:10 despite low circulating B cell counts (CD20^+^ 1.5%, CD19^+^ 1.3%) and normal serum immunoglobulins (IgG 8.54 g/L, IgM 0.35 g/L). Cranial MRI revealed no neuroinflammatory lesion. The patient was given telitacicept 160 mg per week for four weeks and 160 mg per two weeks for subsequent eight weeks, resulting in complete serum anti-LGI1 antibody clearance. Clinical monitoring demonstrated marked resolution of affective symptoms and memory recovery with an mRS score of 1, a CASE score of 2, and an MMSE score of 25. Post-treatment serological test revealed CD20^+^ B cell reduction to 1.1% and CD19^+^ B cell to 1.2%, with preserved humoral immunity (IgG 7.33 g/L, IgM 0.32 g/L).

During the 6-month follow-up, sustained clinical stability was maintained with progressive resolution of mood and sleep disturbance and memory deficit, achieving a MMSE score of 26.

### 2.3 Case 3

A 37-year-old female was admitted for cognitive impairment and myokymia in lower limbs for one month, accompanied by weakness, paresthesia, palpation, and sleep disorder. Physical examination demonstrated impaired arithmetic ability and generalized limbic muscle weakness (muscle strength graded 4-). Functional assessment revealed an mRS score of 3, a CASE score of 4, and an MMSE score of 18. Laboratory studies identified hyponatremia (129 mmol/L). CSF analysis showed normal opening pressure, cell count, proteins, glucose, and chloride levels. Electromyogram showed no abnormalities, yet electroencephalogram depicted mild θ wave activity with individual sharp waves in the left central temporal area. Brain MRI revealed T2-weighted fluid attenuated inversion recovery (T2-FLAIR) and diffusion weighted imaging (DWI) hyperintensity in the right hypothalamus and bilateral medial temporal lobes. ^18^F-FDG-PET/CT demonstrated hypermetabolism in the right hypothalamus and focally in the left occipital lobe. ^18^F-DPA714 PET/MRI showed significantly increased uptake in the bilateral medial temporal lobes (**Figure 1**). The AE-related antibodies panel test (CBA, Simcere Diagnosis, Nanjing, China) confirmed dual positivity for anti-LGI1 antibody (titer CSF 1:30, serum 1:100) and anti-contactin-associated protein 2 (CASPR2) antibody (titer CSF 1:10, serum 1:100).

The patient was diagnosed with anti-LGI1 and anti-CASPR2 positive encephalitis and initiated on IVMP combined with efgartigimod (10 mg/kg per week for a total of four doses) for treatment. Post-treatment serology showed IgG reduction from 12.78 g/L to 4.37 g/L, with complete serum clearance of anti-CASPR2 antibodies and serum anti-LGI1 titer declined to 1:10. Her symptoms improved significantly, with an mRS score of 1, a CASE score of 2, and an MMSE score of 29. Yet, the patient continued to experience nocturnal restless legs syndrome and persistent limb weakness. To further consolidate the therapeutic effect and, more importantly, to accelerate corticosteroid tapering, telitacicept was initiated as sequential therapy with 160 mg every 2 weeks for 8 weeks, together achieving full serological remission (anti-LGI1 negative) and functional recovery (mRS 0, CASE 0). During the treatment period, oral prednisone was tapered by 5 mg every two weeks until reaching a maintenance dose of 5 mg. At the 6-month post-treatment evaluation, longitudinal monitoring demonstrated complete resolution of neurological manifestations, with serological surveillance confirming sustained undetectable antibody titers.

## 3 Discussion

AE is a potentially life-threatening neurological disorder characterized by acute or subacute onset, rapid progression, and plausible relapses (1). While most patients achieve favorable outcomes with immunotherapy, clinical management remains challenging in refractory cases. For patients showing suboptimal response to initial therapy, current guidelines recommend either repeating first-line interventions or escalating to second-line agents (12). Recent years have witnessed expanding therapeutic options with the experimental use of novel monoclonal antibodies such as OFA and efgartigimod, which have demonstrated partial efficacy in selected cases (13, 14). However, a subset of patients develops disease that is refractory to multiple lines of therapy. Furthermore, treatment-related complications, particularly from long-term corticosteroid use, limit therapeutic options for specific patients. These unmet clinical needs underscore the imperative for developing targeted therapies with improved efficacy and safety profiles.

The B cell lineage constitutes a central pathogenic pathway in neuroimmunological disorders, with plasma cells as primary effectors of pathological antibody production. Current AE therapeutics target specific developmental stages of B cell lineage. RTX and OFA induce complement-dependent cytotoxicity against CD20^+^ B cells (15). This mechanism effectively depletes both circulating naive and memory B cell pools, rapidly eliminating pathogenic B cells and reduce nascent antibody-secreting cell sources (3, 16). However, plasma cells naturally do not express CD20 on their surface. As a result, RTX and OFA spares these CD20^-^ antibody-secreting cells, including plasmablasts, short-lived plasma cells (SLPC) and long-lived plasma cells (LLPS) (17). Plasmablasts and SLPCs mediate acute relapses through rapid production of pathogenic antibody, whereas LLPCs establish chronic autoimmunity by sustaining persistent antibody production, thereby underpinning treatment resistance to conventional immunosuppressants (5). This therapeutic gap is clinically significant: a portion of AE patients receiving CD20-targeted therapy experience poor prognosis, manifesting as residual disability or delayed relapses (4).

Telitacicept is a novel recombinant fusion protein comprising the extracellular domain of the human transmembrane activator and calcium-modulating cyclophilin ligand interactor and immunoglobulin Fc fragment. It demonstrates distinct mechanism relative to CD20-targeted agents through its dual inhibition of BLyS and APRIL (18). BLyS is essential for B cell survival, particularly for follicular, marginal zone, and mature subsets (6). More importantly, the survival of plasma cells is dependent on BLyS and APRIL, with a particularly strong reliance on APRIL (6). This bispecific blockade not only inhibits the proliferation of autoreactive B cells but also directly induces apoptosis across the antibody-secreting cells spectrum, including plasmablasts, SLPC and LLPC, thereby eliminating antibody production at its source (19). In March 2021, telitacicept received its first approval in China for treating patients with active SLE. Moreover, emerging evidence has shown its efficacy and safety in myasthenia gravis (9), and a retrospective study also reported remarkable symptomatic improvement in refractory generalized myasthenia gravis (10). These findings provide a rationale for exploring telitacicept in refractory AE.

**Figure 2:**
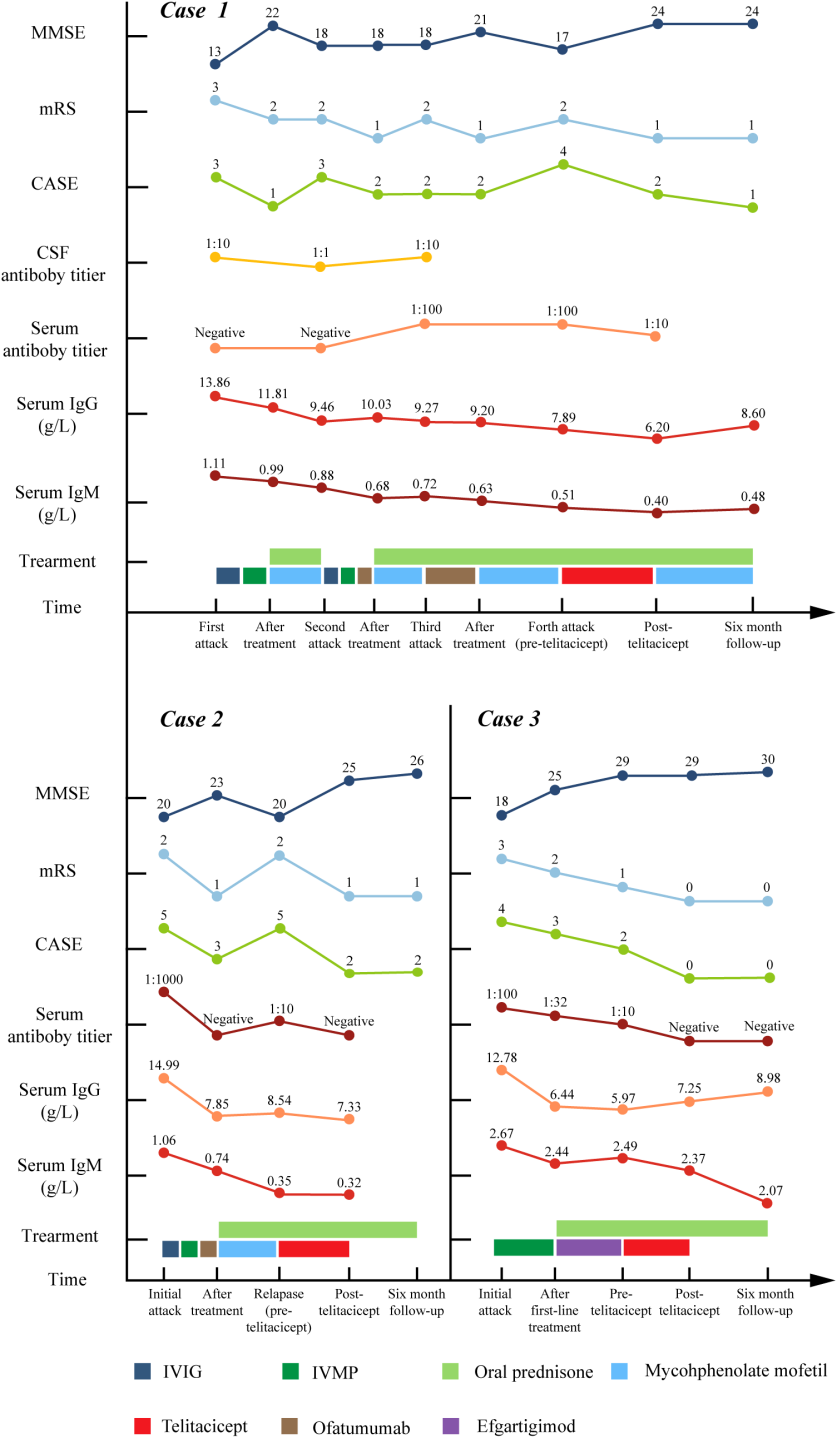
The longitudinal changes of MMSE score, mRS score, CSF and serum antibody titer, serum IgG and IgM level, treatment details in three cases. MMSE, Mini-Mental State Examination; mRS, modified Rankin Scale; CASE, Clinical Assessment of Autoimmune Encephalitis; CSF, cerebrospinal fluid; IVIG, intravenous immunoglobulin; IVMP, intravenous methylprednisolone pulse.

This case series presented three antibody-confirmed AE patients with distinct clinical challenges (**Table 1**, **Figure 2**): (1) Case 1 suffered from repetitive relapses yet the use of repeated methylprednisolone impulse treatment was constrained by severe comorbidities (upper gastrointestinal bleeding); (2) Case 2 was a refractory case demonstrating unsatisfactory response to IVIG, corticosteroids and OFA; (3) Case 3 was a newly-onset dual antibody-positive (anti-LGI1/CASPR2) patient necessitating sustained immunomodulation to facilitating antibody clearance and prednisone tapering. Hence, telitacicept was started for them. In refractory and relapsed cases (Cases 1 and 2), telitacicept demonstrated disease-stabilizing effects with modest clinical improvement. In the patient with a shorter disease course (Case 3), telitacicept served as sequential therapy, accelerating antibody seronegative conversion, enabling corticosteroid tapering, and promoting sustained functional recovery.

**Table 1:** The clinical characteristics of the three patients.

Case No	CASE 1	CASE 2	CASE 3
Age	56	42	37
Sex	Male	Male	Female
Antibody	Anti-NMDAR	Anti-LGI1	Anti-LGI1 Anti-CASPR2
Symptoms	Psychiatric disorder, cognitive impairment, sensory disorder	Seizure, cognitive impairment, mental disorder	Myokymia, cognitive impairment, autonomic dysfunction
The immunotherapy before telitacicept	IVIG, IVMP, OFA, MMF	IVIG, IVMP, OFA, MMF	IVMP, efgartigimod
The titer of antibodies at the initial onset	CSF, 1:10	CSF, 1:10 Serum, 1:1000	Anti-LGI1: CSF, 1:30; Serum, 1:100 Anti-CASPR2: CSF, 1:10; Serum, negative
The titer of antibodies before telitacicept	Serum, 1:100	Serum, 1:10	Anti-LGI1: Serum, 1:10 Anti-CASPR2: Serum, negative
The titer of antibodies after telitacicept	Serum, 1:10	Serum, negative	Anti-LGI1: Serum, negative Anti-CASPR2: Serum, negative
Serum IgG before telitacicept (g/L)	7.89	8.54	5.97
Serum IgG after telitacicept (g/L)	6.2	7.33	7.25
Serum IgM before telitacicept (g/L)	0.51	0.35	2.49
Serum IgM after telitacicept (g/L)	0.40	0.32	2.37
mRS before telitacicept	2	2	1
mRS after telitacicept	1	1	0
mRS at 6-month follow-up	1	1	0
CASE before telitacicept	4	5	2
CASE after telitacicept	2	2	0
CASE at 6-month follow-up	1	2	0
MMSE score before telitacicept	17	20	29
MMSE score after telitacicept	24	25	29
MMSE score at 6-month follow-up	24	26	30

NMDAR, N-methyl-D-aspartate receptor; LGI1, leucine-rich glioma-inactivated 1; CASPR2, contactin-associated protein 2; IVIG, intravenous immunoglobulin; IVMP, intravenous methylprednisolone pulse; OFA, ofatumumab; MMF, mycophenolate mofetil; CSF, cerebrospinal fluid; mRS, modified Rankin Scale; CASE, Clinical Assessment of Autoimmune Encephalitis; MMSE, Mini-Mental State Examination.

To date, only a single case study has documented the application of telitacicept for AE treatment. This report described its successful use as sequential therapy in a patient with overlapping MOGAD and anti-NMDAR encephalitis (11). Our study encompasses a broader range of antibody types, including not only anti-NMDAR antibody but also anti-LGI1 and anti-CASPR2 antibody. Unlike the referenced study where RTX was used for just one treatment course and showed good efficacy, our patient in Case 1 experienced poor disease control despite multiple rounds of OFA therapy, prompting our switch to telitacicept.

Notably, in Cases 1 and 2, their previous treatment of OFA had induced partial remission, evidenced by the low levels of circulating CD20+ B cells. However, disease relapsed despite ongoing OFA treatment, implying the possible involvement of CD20^-^ plasma cells in the relapse pathogenesis. A recent study on refractory lupus nephritis demonstrated all 7 patients in the RTX plus telitacicept group achieving complete renal response, while only 76% of patients received RTX monotherapy attained the same outcome (20). These results highlighted the unique mechanism advantage of telitacicept, and provided evidence for telitacicept as a supplement to CD20-targeted therapy.

Glucocorticoids serve as the cornerstone of first-line treatment for AE. However, high doses or prolonged use can lead to severe cumulative side effects, including increased infection risks, osteoporosis, hypertension, cataracts, and metabolic disturbance (21). A significant proportion of AE patients, particularly those with refractory or recurrent cases, require long-term oral prednisone maintenance therapy to prevent relapse, making dose reduction challenging. While immunomodulatory agents like AZA and MMF are used for long-term maintenance immunotherapy and demonstrate some steroid-sparing effects (22), their utility is limited by slow onset, interindividual variability, and safety concerns (e.g., bone marrow suppression, hepatotoxicity, infection risk) (23). There is thus an urgent need to identify immunomodulatory strategies that maintain disease control while enabling effective steroid dose reduction with improved safety profiles. Clinical data from myasthenia gravis studies show that telitacicept significantly lowers steroid dosage at 2 months and 4 months after baseline, with some patients achieving complete steroid discontinuation (24). In Case 3, telitacicept was used as sequential therapy to accelerate steroid tapering while maintaining disease stability. These findings provide mechanistic and preliminary clinical support for its potential in steroid reduction for AE.

Telitacicept exhibits a favorable safety profile, with only transient injection reactions and an absence of severe infections observed in our cases. While anti-CD20 agents causes profound B cell exhaustion which increases infection risks, telitacicept modulates B cell maturation without eradicating the entire B cell pool, preserving regenerative capacity and selectively sparing APRIL-independent long-lived plasma cells to maintain protective immunity. This has been partially confirmed in our cases, as the immunoglobulin level of all three patients was well preserved above the lower limit.

In the phase 2 clinical trial, adverse events were observed in 64.3% of patients in the telitacicept 160 mg group and 80.0% of patients in the 240 mg group, but most of them were of mild to moderate severity (9). Infections (predominantly upper respiratory tract infections and urinary tract infections), blood immunoglobulin decrease (IgG, IgA, and IgM), and diarrhea are the most common treatment-emergent adverse events (8, 10). Our patient did not experience any significant adverse events during the six-month observation period, potentially attributed to the mild dosage used in our cases compared to other studies. Our patient in Case 1 developed upper gastrointestinal bleeding during corticosteroid treatment but there is currently no evidence suggesting that telitacicept causes upper gastrointestinal bleeding. And our patient experienced no related adverse events during six-month observation.

The tolerability highlights telitacicept’s promising potential in patients with infection risks and comorbidities. Severe infections represent one of the most frequent direct causes of adverse event-related mortality in AE (25, 26). This risk is particularly significant for elderly patients and those with comorbidities such as diabetes or chronic lung disease, necessitating more cautious use of immunosuppressive agents in these populations. Additionally, Telitacicept’s weekly subcutaneous administration offers significant adherence advantages over intravenous alternatives (rituximab/IVIG) or daily oral regimens. This is particularly beneficial for elderly patients and those with mobility limitations who face challenges with clinic-based infusions or complex medication schedules.

To conclude, this case series proposes telitacicept as a promising therapeutic alternative for AE through BLyS/APRIL dual-pathway inhibition. Our findings support its further evaluation as a second-line or rescue agent in refractory AE and as a sequential therapy to reduce steroid dependence. Nevertheless, our study’s generalizability is limited by its small sample size and short-term follow-up duration. The concomitant use of prednisone and mycophenolate mofetil should also be considered as potential confounding factors. The preliminary observations necessitate validation through large-scale multicenter studies with extended observation periods to conclusively establish telitacicept’s therapeutic efficacy and relapse-prevention capacity in heterogeneous AE populations.

## 4 Patient perspective

All the three patients were fully informed and adhered to the treatment regimen throughout the clinical course. Patient 1 expressed significant distress over previous disease relapses and approached the new treatment with caution due to a history of corticosteroid-induced gastrointestinal bleeding. Following Telitacicept therapy, his symptoms alleviated, and both the patient and his family were very glad for the clinical improvement. Patient 2 reported that his most recent relapse necessitated temporary work leave. He conveyed satisfaction with Telitacicept treatment, noting his successful return to employment. Patient 3 was reluctant about long-term corticosteroid use. She reported satisfaction with Telitacicept for enabling corticosteroid dosage reduction. All patients provided consent for publication of their anonymized case details.

## Data Availability

The original contributions presented in the study are included in the article/Supplementary Material. Further inquiries can be directed to the corresponding author/s.
